# Case Report: A Young Man With Giant Pericardial Synovial Sarcoma

**DOI:** 10.3389/fcvm.2022.829328

**Published:** 2022-01-27

**Authors:** Yong Luo, Ke Gong, Ting Xie, Ruilin Liu, Hui Guo, Lei Wang, Zhiping Tan, Shi jun Hu, Yifeng Yang, Li Xie

**Affiliations:** ^1^Department of Cardiovascular Surgery, The Second Xiangya Hospital of Central South University, Central South University, Changsha, China; ^2^The State Key Laboratory of Medical Genetics, The Clinical Center for Gene Diagnosis and Therapy, The Second Xiangya Hospital of Central South University, Central South University, Changsha, China

**Keywords:** pericardial synovial sarcomas, pericardium, tumor, treatment, prognosis

## Abstract

Pericardial synovial sarcomas are sporadic tumors. Herein, we report a case of primary pericardial synovial sarcoma originating from the right pericardium. Missed diagnosis delayed surgical treatment. Eventually, the tumor occupied the almost entire pericardial cavity. The pericardial tumor was surgically removed as soon as possible after admission. In this paper, we aim to provide details that can help further understand the differing symptoms and presentations of pericardial synovial sarcoma and highlight the importance of consideration of this disease in similar cases where the etiology of pericardial effusion is unknown.

## Introduction

Pericardial synovial sarcoma (PSS) is a rare malignant tumor. Thus far, only a few cases of PSS have been reported. Synovial sarcoma (SS) is also reported in some uncommon sites such as the pleura, heart, kidney, lung, prostate, liver, gastrointestinal tract, and peripheral nerves. The diagnosis of SS in these unusual locations is challenging and requires additional diagnostic procedures such as immunohistochemistry, electron microscopy, and molecular genetic techniques for confirmation of the diagnosis. The case cardiac tamponade presented as the initial symptom, but no obvious evidence supported the diagnosis of pericardial synovial sarcoma. After 5 months, the anterior mediastinal tumor was found using transthoracic echocardiography. Non-random occurrence of t(x;18) was consistently found in SS. In some cases, the only cytogenetic abnormality noted is the t(X;18), suggesting that this is a key molecular event in tumor development (1). The survival rate of patients with primary cardiac sarcoma is poor, and most patients die within a few months after the diagnosis is confirmed. However, because of the rarity of PSS, it is difficult to determine the prognostic factors; however, diagnosis at a young age, lack of complicated chromosome abnormalities, and tumor originating from the pericardium appear to be factors pointing to good prognosis (2).

## Case Report

**Figure 1:**
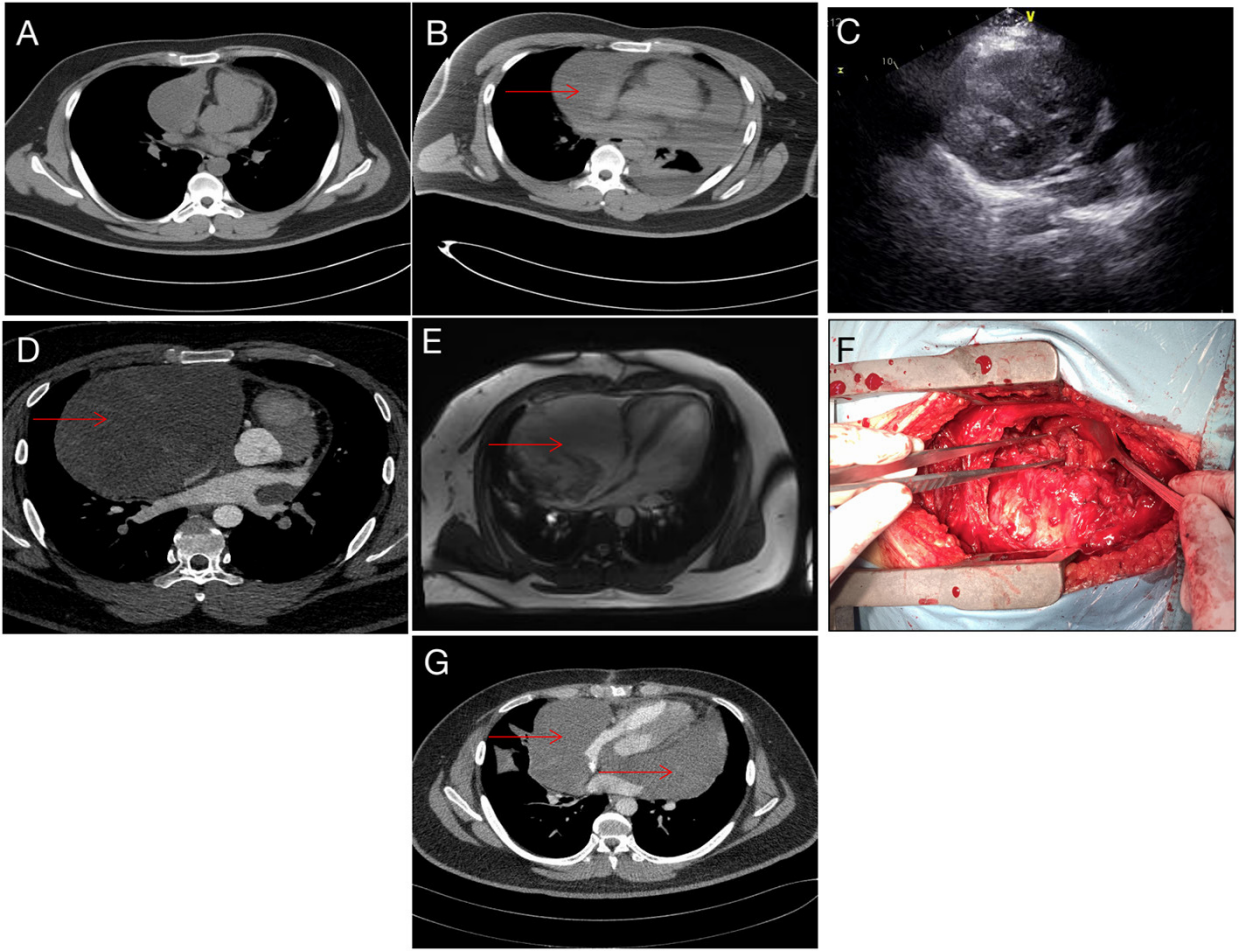
Imaging data. **(A)** CT image taken during the first admission. **(B)** After pericardiocentesis, CT indicating a suspicious mass (red arrow). **(C)** Echocardiography showing pericardial space-occupying lesions. **(D)** Reexamination after 5 months showed obvious space occupation (red arrow). **(E)** Preoperative magnetic resonance imaging, right pericardium, showing a cystic mass (red arrow). **(F)** During the operation, the tumor filled the pericardial cavity. **(G)** Tumor recurrence noted 5 months after operation (red arrow).

**Figure 2:**
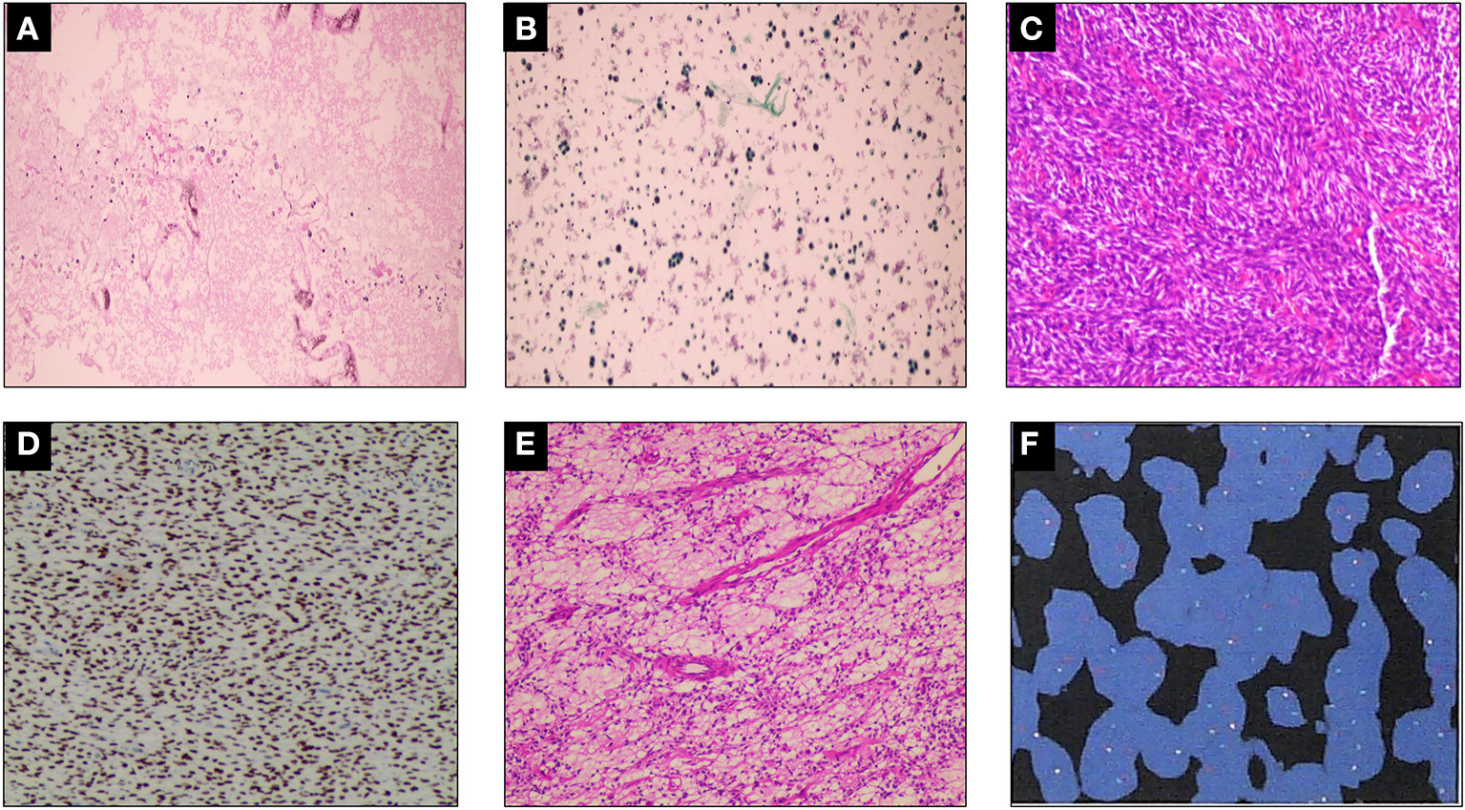
Histopathological examination. **(A)** A small number of mesothelial cells and lymphocytes were found in the pleural effusion, but no malignant tumor cells were found. **(B)** Tissue cells, lymphocytes, and no malignant tumor cells could be found in the left pleural effusion. *Mycobacterium tuberculosis* was found to be negative by acid-fast staining (–). **(C–E)** Damaged tissue of pericardial tumor, which measured 20 × 17 × 5 cm, with some gelatinous areas. Mesenchymal tissue-derived tumors, with dense spindle cell areas and edema areas, combined with immunohistochemistry, do not rule out SS. Immunohistochemistry results: CK (–), EMA (focal +), TLEL (+), CD34 (–), S100 (–), CD99 (partial +), Bcl-2 (+), Ki67 (hot spot 25% +), WT-1 (–), Desmin (–), SMA (–), Vim (+), calponin (partial +), INI-1 (–), ERG (–), p53 (30% weak +), HMB45 (–), FLI-1 (–). **(F)** Fluorescence *in situ* hybridization (FISH) showing rearrangement of the SS18 gene, indicating positive result.

A 19-year-old man was referred to our hospital for the first time because of chest tightness and dyspnea. Chest echocardiography and computed tomography (CT) revealed a large amount of pericardial effusion, pleural effusion, left lung swelling, and right lower lung swelling (Figure 1A). The patient was subjected to pericardiocentesis and drainage; the drained fluid was sent for pathological examination. The pathology report revealed a lack of tumor cells (Figure 2A); acid-fast staining of the sample was also not positive for *Mycobacterium tuberculosis* (Figure 2B). The patient was also subjected to bone marrow puncture; however, the biopsy sample did not show any positive results. Following pericardiocentesis and treatment aimed at providing symptomatic relief, his pleural effusion and pericardial effusion were absorbed (Figure 1B). He was then discharged after symptom improvement. Therefore, some special manifestations of patients have been ignored. Although a mediastinal mass was noted in the first CT images, the pathological examination and related auxiliary examinations of pericardial effusion did not discover any positive results (Figure 1B). Five months later, the patient was re-examined at the local hospital and was found to have a pericardial mass through echocardiography and chest CT.

Then the patient had seen a doctor again for further treatment. Echocardiography, CT, and nuclear magnetic resonance imaging (MRI) at our hospital indicated that the right anterior mediastinum mass had compressed the right heart (Figure 1C) and the aortic root; it measured 15.3 × 11.7 × 15.1 cm (Figures 1D,E). He had no family history relevant to cardiac complaints. Following relevant preoperative examinations, he underwent mass resection *via* a median sternum incision. The size of the mass was about 20 × 15 × 19 cm (Figure 1F). The mass was found to completely cover the right atrium, superior and inferior vena cava, ascending aorta, and part of the pulmonary artery; it also partly covered the anterior wall of the right ventricle. After the operation, pathological examination (Figures 2C–E); furthermore, an SS18 gene test was done (Figure 2F). It was found that the SS18 gene was broken, so the result was positive, suggesting SS. A postoperative echocardiography performed 2 months after discharge demonstrated a heterogeneous hypoechoic lump in the pericardium; thus, tumor recurrence was considered. After 2 months of treatment with traditional Chinese medicine, he was referred to our hospital again. CT angiography revealed a more significant layer of cystic consolidation on the left margin of the heart that measured about 12 × 6.9 cm (left), 11.5 × 5.6 cm (right) (Figure 1G). Thus, following discussion with the patient, chemotherapy was initiated. The patient is currently undergoing chemotherapy with regimen at the oncology department of our hospital.

## Discussion

The incidence of heart tumors is quite hidden, and most of them are caused by accidentally discovered cardiac lumps. These occasional phenomena often means thrombus or neoplasms, which usually occur in specific clinical environments (3). The incidence rate of cardiac tumors ranges from 0.001 to 0.03%. The overwhelming majority of these tumors are benign, and only about one-fourth are malignant. PSS is an even rarer form of cardiac tumors. As of 2018, only 36 cases have been reported in the literature; thus, limited data prevent further understanding of this condition (4). However, this lesion undoubtedly predicts a dismal prognosis (5).

SS is an uncommon malignant tumor mainly seen in children and adolescents; its 10-year overall survival rate is about 0–20% (6, 7). The name SS is possibly indicative of its origin in synovial tissue. It has a biphasic appearance on histology and is divided into two different subtypes: spindle and epithelial cell subtypes. However, no epithelioid differentiation was found in tumor tissues, neither were epithelial markers, such as IHC staining of cytokeratin, observed. Therefore, the real origin of SS remains unknown. The characteristic chromosomal abnormality of SS is t(X;18) (p11.2; q11.2). This has a sensitivity of about 90–100%, strongly suggesting its direct involvement in certain aspects of the occurrence and progression of this cancer (1, 8). Even in our case, the diagnosis of SS was proven following a positive result of the SS18 genetic testing.

The morbidity rate of young people with PSS is relatively high, and the most common site of the disease is in the pericardium (9). Even in our case, the tumor originated from the pericardium. The clinical symptoms of the disease are non-specific, with dyspnea and chest pain being the most common presenting symptoms. The patient described herein presented with chest tightness, dyspnea, and fatigue before admission. There was a large amount of pericardial effusion noted in the early stage. According to a new study conducted in China, the most common causes of pericardial effusion were found to be malignant tumors and tuberculosis (10). However, in our case, pericardiocentesis and etiological examination of the pleural effusion did not support the diagnosis of malignant pleural effusion. This could be possible because malignant tumor cells may not be present in 25% of the cases of malignant pericardial effusion (10). Therefore, despite the absence of malignant cells in the pericardial effusion, the possibility of malignant pleural effusion should not be completely ruled out.

The reason for the initially missed diagnosis in our case was the absence of tumor cells and related markers in the pleural fluid. Besides, the patient's symptoms improved significantly after symptomatic treatment. However, the initial beginning stage of the tumor can be seen on the CT performed initially. After 5 months, the tumor had grown up, leading to aggravation of the patient's condition. It was visible on echocardiography and compressed the right heart and the root of the great artery.

As regards the treatment of PSS, on the one hand, due to the invasiveness and expansibility of the tumor, it is generally impossible to remove the tumor completely. On the other hand, there is no systemic study on this topic due to extremely low incidence of the disease; thus, no optimal treatment strategy has yet been proposed. Besides surgical resection, radiotherapy and chemotherapy are also used; this, however, depends on the clinician's discretion and the patient's condition. In general, surgical resection should be performed as soon as possible once the tumor diagnosis is confirmed, especially when the possibility of malignancy is high. Resection can be performed based on the preoperative MRI evaluation, with the premise of negative margin. Because positive margin is an independent risk factor for cardiac tumors, we recommend MRI before surgery to evaluate the degree of disease and assess whether it can be resected on the premise of negative margin. If this is not possible, second-line radiotherapy and chemotherapy can be considered; evaluation can then be performed and resection undertaken after the tumor has shrunk in size until it can be resected with a negative margin (11). Reports of successful total artificial heart transplantation have been published, and this can also be a solution (12). Although patients with PSS have a low probability of lung metastasis, according to the evaluation by Lee et al., metastatic resection is a good choice for metastatic SS (13). In a recent study, a comparative result of the treatment of primary cardiac sarcoma was reported. Although some authors found no improvement in survival rate compared with traditional surgical treatment (14, 15). However, other authors have found that many patients survive longer after transplantation (16). The patient described herein also considered heart transplantation but considered chemotherapy first due to the lack of donors.

As regards prognosis, a recent statistical study showed that the 2-year survival rate following complete surgical resection of PSS was 75.2% (± 9.7 SE), while that following incomplete resection was 55.0% (± 11.5SE) (Log-Rank test: *P* = 0.029). The survival rate at 12 months was 96.5% (±3.8 SE) in patients treated with chemoradiotherapy (CRT), while it was 21.9% (±10.8 SE) in those not treated with CRT (Log-Rank test: *P* ≤ 0.0001) (17). Thus, it was concluded that advanced age and absence of CRT were significantly associated with reduced survival, while total tumor resection was associated with remarkably improved survival. Furthermore, the clinical outcomes of patients receiving postoperative adjuvant chemotherapy was good, and adjuvant chemotherapy was found to be associated with significantly prolonged survival (18–22). Putnam et al. showed that radiotherapy alone seemed to have little impact on prognosis (23). The age of patients with PSS was also shown to be a strong prognostic indicator in this study. In a large meta-analysis of 1,268 patients with SS, Sultan et al. found that younger patients had better outcomes than older patients; (24) this finding was also confirmed by other authors (25–27). The patient in our report was 19 years old; thus, this young age is indicative of a favorable prognosis. After surgical resection, the patient relapsed 2 months later and was transferred to the Department of Oncology for chemotherapy.

## Conclusion

Herein, we describe a case of a patient with PSS. The missed diagnosis highlights the necessity and difficulty of differential diagnosis in patients with such tumors. Histopathology is key to the diagnosis of PSS. In our case, no abnormality in the pericardial effusion was noted, and our patient presented with atypical clinical symptoms. Although primary PSS is not common, when the etiological diagnosis of pericardial effusion is unknown, further tests need to be carried out to help rule out or confirm the diagnosis of PSS.

## Data Availability Statement

The raw data supporting the conclusions of this article will be made available by the authors, without undue reservation.

## Ethics Statement

Written informed consent was obtained from the individual(s) for the publication of any potentially identifiable images or data included in this article.

## Author Contributions

YL, ZT, and LX contributed to conception and design of the study. YL completed the data collection and wrote the first draft of the manuscript. YL, KG, TX, HG, RL, SH, and LW wrote sections of the manuscript. All authors contributed to manuscript revision, read, and approved the submitted version.

## Funding

This study was supported by the National Science Foundation for Young Scientists of China (8150020951) and the Natural Science Foundation for Young Scientists of Hunan Province (2016JJ4099).

## Conflict of Interest

The authors declare that the research was conducted in the absence of any commercial or financial relationships that could be construed as a potential conflict of interest.

## Publisher's Note

All claims expressed in this article are solely those of the authors and do not necessarily represent those of their affiliated organizations, or those of the publisher, the editors and the reviewers. Any product that may be evaluated in this article, or claim that may be made by its manufacturer, is not guaranteed or endorsed by the publisher.
